# Exploring hub pyroptosis-related genes, molecular subtypes, and potential drugs in ankylosing spondylitis by comprehensive bioinformatics analysis and molecular docking

**DOI:** 10.1186/s12891-023-06664-8

**Published:** 2023-06-29

**Authors:** Xin Li, Xiangying Li, Hongqiang Wang, Xiang Zhao

**Affiliations:** 1 Henan University People’s Hospital, Henan Provincial People’s Hospital, Zhengzhou, China; 2 Department of Surgery of Spine and Spinal Cord, Henan Provincial People’s Hospital, Henan International Joint Laboratory of Intelligentized Orthopedics Innovation and Transformation, Henan Key Laboratory for Intelligent Precision Orthopedics, People’s Hospital of Zhengzhou University, People’s Hospital of Henan University, Zhengzhou, China

**Keywords:** Ankylosing spondylitis, Pyroptosis, Molecular subtype, Machine learning, GZMB

## Abstract

**Background:**

Ankylosing spondylitis (AS) is a chronic inflammatory autoimmune disease, and the diagnosis and treatment of AS have been limited because its pathogenesis is still unclear. Pyroptosis is a proinflammatory type of cell death that plays an important role in the immune system. However, the relationship between pyroptosis genes and AS has never been elucidated.

**Methods:**

GSE73754, GSE25101, and GSE221786 datasets were collected from the Gene Expression Omnibus (GEO) database. Differentially expressed pyroptosis-related genes (DE-PRGs) were identified by R software. Machine learning and PPI networks were used to screen key genes to construct a diagnostic model of AS. AS patients were clustered into different pyroptosis subtypes according to DE-PRGs using consensus cluster analysis and validated using principal component analysis (PCA). WGCNA was used for screening hub gene modules between two subtypes. Gene Ontology (GO) terms and Kyoto Encyclopedia of Genes and Genomes (KEGG) pathways were used for enrichment analysis to elucidate underlying mechanisms. The ESTIMATE and CIBERSORT algorithms were used to reveal immune signatures. The connectivity map (CMAP) database was used to predict potential drugs for the treatment of AS. Molecular docking was used to calculate the binding affinity between potential drugs and the hub gene.

**Results:**

Sixteen DE-PRGs were detected in AS compared to healthy controls, and some of these genes showed a significant correlation with immune cells such as neutrophils, CD8 + T cells, and resting NK cells. Enrichment analysis showed that DE-PRGs were mainly related to pyroptosis, IL-1β, and TNF signaling pathways. The key genes (TNF, NLRC4, and GZMB) screened by machine learning and the protein–protein interaction (PPI) network were used to establish the diagnostic model of AS. ROC analysis showed that the diagnostic model had good diagnostic properties in GSE73754 (AUC: 0.881), GSE25101 (AUC: 0.797), and GSE221786 (AUC: 0.713). Using 16 DE-PRGs, AS patients were divided into C1 and C2 subtypes, and these two subtypes showed significant differences in immune infiltration. A key gene module was identified from the two subtypes using WGCNA, and enrichment analysis suggested that the module was mainly related to immune function. Three potential drugs, including ascorbic acid, RO 90–7501, and celastrol, were selected based on CMAP analysis. Cytoscape showed GZMB as the highest-scoring hub gene. Finally, molecular docking results showed that GZMB and ascorbic acid formed three hydrogen bonds, including ARG-41, LYS-40, and HIS-57 (affinity: -5.3 kcal/mol). GZMB and RO-90–7501 formed one hydrogen bond, including CYS-136 (affinity: -8.8 kcal/mol). GZMB and celastrol formed three hydrogen bonds, including TYR-94, HIS-57, and LYS-40 (affinity: -9.4 kcal/mol).

**Conclusions:**

Our research systematically analyzed the relationship between pyroptosis and AS. Pyroptosis may play an essential role in the immune microenvironment of AS. Our findings will contribute to a further understanding of the pathogenesis of AS.

**Supplementary Information:**

The online version contains supplementary material available at 10.1186/s12891-023-06664-8.

## Background

Ankylosing spondylitis (AS) is an immune-mediated inflammatory disease with a strong genetic predisposition that affects 0.1%-0.3% of the adult population worldwide [1, 2]. AS is generally characterized by inflammatory damage to the axial skeleton such as the sacroiliac joints and spinal joints [3]. Patients mainly present with chronic low back pain and decreased spinal mobility [4]. As the disease progresses, it can lead to spinal ankylosis, spinal deformity, and even disability, which significantly reduces the patient’s quality of life [5]. Currently, the pathogenesis of AS is not fully understood, leading to a lack of biomarkers with high diagnostic value for AS [6]. Recent studies suggest that HLA-B27 is closely related to AS and that the rate of HLA-B27 positivity in AS patients is as high as 90%. Unfortunately, the specificity of HLA-B27 is low, and only 5% of HLA-B27-positive individuals have AS [7]. Therefore, the diagnosis of AS remains inaccurate until imaging suggests sacroiliac arthritis. In the treatment of AS, with the advent of tumor necrosis factor inhibitors (TNFi), interleukin-17 inhibitors, and Janus kinase inhibitors, patients with AS have seen a dramatic improvement in symptom control [8]. TNFi, in particular, has been widely used in patients with an inadequate response to NSAIDs. However, TNFi is not effective in all patients, and it has been reported in the literature that approximately half of the patients do not have significant improvement in symptoms after the application of TNFi, suggesting a large heterogeneity in drug response in AS patients [9–11]. Therefore, it is important to further explore the pathogenesis of AS and the appropriate molecular subtypes.

Currently, it is believed that dysfunction of the body’s immune system plays a critical driving role in the pathological progression of AS and that immune cells and innate cytokines are closely associated with the pathogenesis of AS [12–14]. Pyroptosis is an inflammatory programmed cell death mode characterized by mediating cell membrane destruction and releasing various cytokines, ultimately promoting inflammatory effects or immune responses [15, 16]. Gasdermin D (GSDMD) protein is the key executor of pyroptosis, which can be cleaved by active caspase-1 and release the N-terminal effector domain, thereby initiating pyroptosis [17]. The NOD-like receptor family pyrin domain containing 3 (NLRP3) inflammasome is an important activator of caspase-1 and plays a key role in the pathogenesis of autoinflammatory and arthritic diseases [18]. Previous studies have shown that the expression of NLRP3 and caspase-1 is upregulated in AS patients [19], suggesting that the activation of the pyroptosis-associated NLRP3 inflammasome may be associated with AS. Therefore, we infer that pyroptosis may regulate the pathological progression of AS through inflammatory injury or cytotoxic effects. However, currently, the study of pyroptosis-related genes in AS is still lacking, and their potential as therapeutic targets for AS remains to be understood.

Our study is the first to comprehensively explore the immune infiltration and molecular functions of differentially expressed pyroptosis-related genes (DE-PRGs) in AS and construct a diagnostic model of AS using machine learning and a PPI network. Subsequently, we identified two pyroptosis-associated clusters with distinct immune signatures using consensus clustering analysis. Finally, in combination with disease differential genes and subtype differential genes, we screened three small-molecule drugs and used molecular docking to analyze the proteins of the hub gene and the potential binding targets and binding ability of small-molecule compounds and provide new insights into the treatment of AS.

## Methods

### Data collection

The Gene Expression Omnibus (GEO) database is the largest fully public gene expression resource that collects gene expression data for multiple species [20]. The GEO database was searched for "Ankylosing spondylitis", set the species to human, and selected Entry type to Series. A total of 33 datasets were eventually retrieved. In order to ensure the quality of the data, we checked each of these datasets for information related to the experimental sample, experimental design, and data type. Finally, three datasets (GSE73754, GSE25101 and GSE221786) were identified from the GEO database [21, 22]. GSE73754 (Microarray, platform GPL10558) included gene expression profiles in whole blood samples from 52 AS patients and 20 healthy controls and was used as a training cohort. GSE25101 (Microarray, platform GPL6947) included gene expression profiles of whole blood samples from 12 AS patients and 12 healthy controls, and GSE221786 (RNA-seq, platform GPL24676) included gene expression profiles of whole blood samples from 20 AS patients and 8 healthy controls, GSE25101 and GSE221786 were used to validate the value of the diagnostic model. Pyroptosis-related genes were derived from the Molecular Signatures Database (MSigDB, version 7.5.1) and are listed in Supplementary file 2.

### Screening for differentially expressed genes

The Illumina platform used by GSE73754 provides a standardized matrix file of microarray data. The “limma” package was used to determine differentially expressed genes (DEGs) between 52 AS samples and 20 healthy controls in GSE73754 with inclusion criteria of *p* < 0.05 and |logFC|> 0 [23]. Subsequently, we determined the DE-PRGs by overlapping DEGs and pyroptosis-related genes (PRGs). The expression levels of DE-PRGs between AS and healthy controls were visualized using the “ggplot2” and “pheatmap” R packages. Significant correlations between DE-PRGs were visualized by the “corrplot” and “circlize” packages.

### Functional enrichment analysis

GO terms and KEGG pathways are commonly used methods in gene enrichment analysis [24]. To understand the biological functions of genes, we performed GO terms and KEGG pathways using the “ClusterProfiler” R package [25].

### Immune infiltration analysis

In this study, we used two algorithms for immune analysis. ESTIMATE was used to calculate the total immune scores of different groups. The CIBERSORT algorithm was used to evaluate the relative proportion of 22 types of immune cell infiltration for each sample (perm = 1000), and the results were filtered according to *p* < 0.05.

Furthermore, we analyzed the correlation between DE-PRGs and immune cell infiltration in AS. We removed healthy controls, extracted the expression of DE-PRGs, and performed correlation analysis with the results of immune infiltration using Pearson’s correlation coefficient. Finally, the results are presented using the “ggplot2” package.

### PPI network construction

The STRING database (https://string-db.org/) is a platform for exploring protein interactions, both physical interactions and functional associations [26]. We use the STRING database to build PPI networks, and a confidence score of 0.4 was set as the cutoff standard. CytoHubba was used to screen key genes in the PPI network.

### Machine learning

Based on the expression of DE-PRGs, least absolute shrinkage and selection operator (LASSO) and random forest (RF) analyses were performed to identify the most characteristic diagnosis-related DE-PRGs. The Lasso model was performed using the “glmnet” package with the penalty parameter (λ) by tenfold cross-validation, the response type set as binomial, and the alpha set as 1. The “randomforest” package was used for RF modeling, and the number of decision trees was set to 500.

### Construction and evaluation of the diagnostic model and nomogram

We used the overlapping genes of the LASSO, RF, and PPI networks to build a diagnostic model and the “rms” package to construct the nomogram and calibration curve. The calibration curve can intuitively demonstrate the prediction ability of the nomogram. Decision curve analysis (DCA) generated with the “rmda” package was used to obtain the highest net benefit and determine the clinical practicality of the nomogram. Finally, receiver operating characteristic (ROC) curve analysis was performed on the GSE73754, GSE25101, and GSE221786 datasets through the “pROC” package [27]. The area under the curve (AUC) was calculated to validate the diagnostic value of the diagnostic model.

### Cluster analysis of AS patients

Based on DE-PRG expression profiles, 52 AS patients were reclassified using the “ConsensusClusterPlus” package (reps = 1000, pItem = 0.8, pFeature = 1, K-means algorithm, distance = euclidean, maxK = 9) [28]. The optimal number of clusters can be determined from consensus matrices, tracking plots, consensus cumulative distribution function (CDF) plots, and delta areas. Finally, differences in the expression of DE-PRGs and the abundance of immune infiltration in different clusters were also clarified.

### Weighted gene correlation network analysis (WGCNA)

The variance was calculated based on the expression of genes in different clusters, and the top 25% of genes were selected for WGCNA [29]. We used the pickSoftThreshold function to analyze and test t soft-threshold power from 1 to 20. Subsequently, an adjacency matrix was generated using the optimal soft-threshold power and transformed into a topological overlap matrix (TOM). Genes with coexpression relationships were grouped using average linkage hierarchical clustering based on the dissimilarity measure (1-TOM). We set the minimum module size to 50 and used the dynamic tree-cut algorithm to identify modules. Module eigengene (ME), as each module’s first principal component, represented each module’s expression pattern. The correlation between each module eigengene and cluster were calculated, and the module with the greatest correlation with the cluster was selected for subsequent analysis.

### The prediction of potential small-molecule drugs

The Connectivity Map (CMAP) database (https://clue.io/) is a drug discovery tool for exploring potential biological associations between diseases, genes, and drugs. By uploading differential genes to CMAP, small molecular drugs that may induce or reverse the biological process of differential gene expression are predicted. The score of the final analysis result is calculated from -100 to 100, and small-molecule drugs with negative scores indicate the potential to reverse gene expression, representing potential therapeutic value. We selected the three small-molecule drugs with the smallest fractions for subsequent analysis.

### Molecular docking

We used the molecular docking method to explore the potential drug targets and binding ability of three small-molecule drugs and hub gene proteins [30]. PubChem was used to obtain the molecular structures of small-molecule drugs. The PDB database can obtain the protein structure of hub genes. Subsequently, PyMOL was used to remove water molecules and ligands in the protein structure. AutoDockTools was used to process ligands and receptors further, mainly by adding hydrogen atoms and determining active pockets. AutoDock Vina software was used to analyze the binding modes between the candidate protein and three small-molecule drugs. Each docking event produced 20 different binding modes, and the model with the lowest binding free energy was finally selected and visualized by PyMOL.

## Results

### Screening differentially expressed genes

**Fig. 1:**
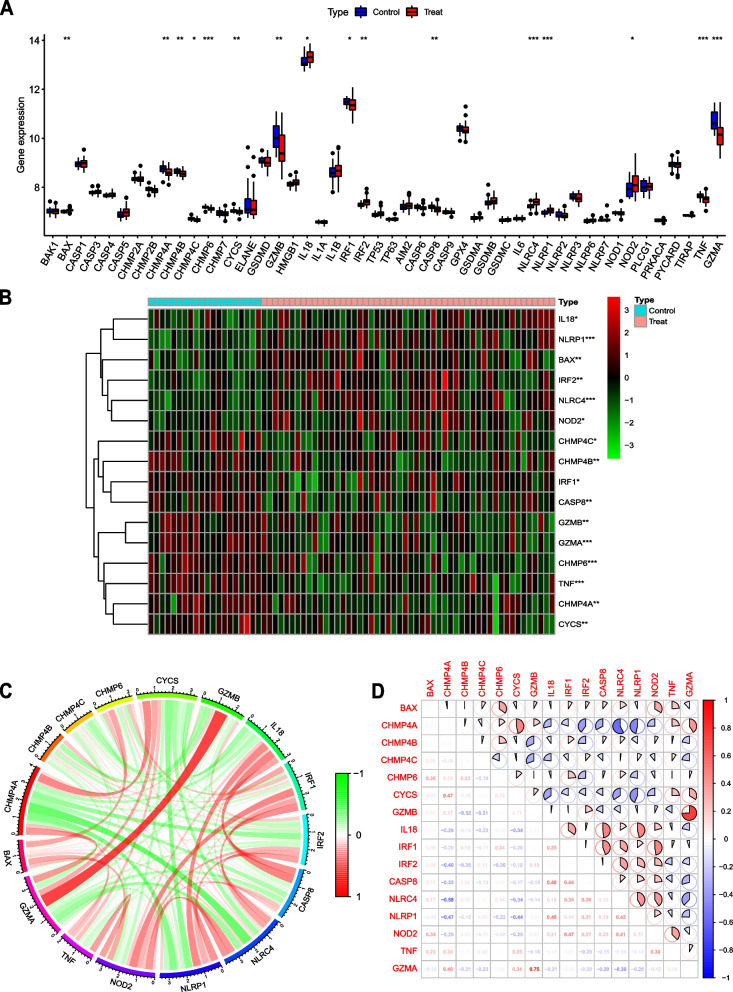
Identification of DE-PRGs in AS. **A** Expression of PRGs in AS and control groups. Blue represents the control group, and red represents the AS group. A total of 52 PRGs were included, 48 PRGs were detected to be expressed in the samples, and 16 PRGs were differentially expressed. *p < 0.05, **p < 0.01, ***p < 0.001. **B** Heatmap showing the expression of the DE-PRGs in AS and control samples. **C** The relationship circle diagram of 16 DE-PRGs. **D** Correlation analysis of 16 DE-PRGs. Blue represents negative correlations, and red represents positive correlations. Correlation coefficients are shown in pie charts and figures

After data preprocessing, we screened 5868 DEGs from the GSE73754 dataset. Subsequently, through a comparison of the expression of 52 PRGs of AS and healthy controls, a total of 16 genes were determined to be differentially expressed. Of these genes, the expression of BAX, IL18, IRF2, NLRC4, NLRP1, and NOD2 in AS blood samples was significantly higher than that in healthy control blood samples, and the expression of CHMP4A, CHMP4B, CHMP4C, CHMP6, CYCS, GZMB, IRF1, CASP8, TNF, and GZMA in AS blood samples was significantly lower than that in healthy control blood samples (Figs. 1A–B). To further understand the relationship between the 16 DE-PRGs, we analyzed the correlation between these genes (Figs. 1C–D). The results revealed that GZMA and GZMB showed an intense synergistic effect (*R* = 0.75), and CHMP4A and NLRC4 presented an apparent antagonistic action (*R* = -0.58).

### Immune infiltration analysis of AS and DE-PRGs

We obtained the infiltration abundance of immune cells in AS and healthy samples using the CIBERSORT algorithm (Supplementary file 3). The obtained results showed that the infiltration levels of neutrophils, regulatory T cells, and naive CD4 + T cells were significantly higher in AS. The infiltration levels of CD8 + T cells, activated memory CD4 T cells, and resting NK cells were significantly lower in AS.

**Fig. 2:**
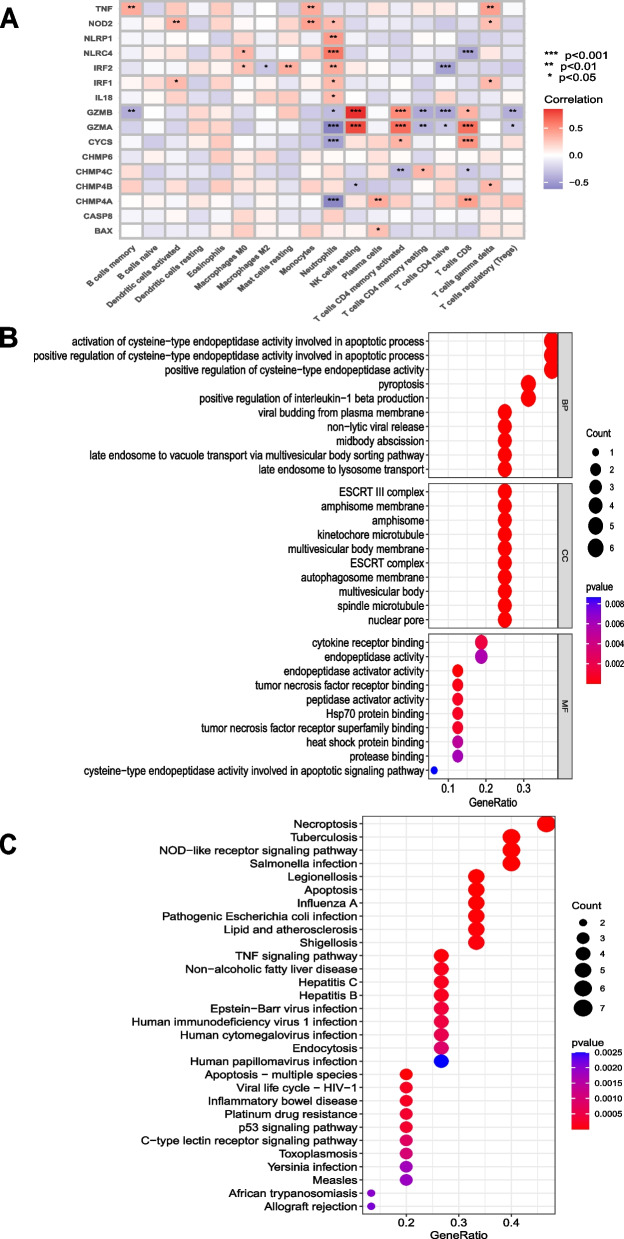
Immune infiltration analysis and enrichment analysis of 16 DE-PRGs. **A** Correlation of DE-PRGs with immune cells. *p < 0.05, **p < 0.01, ***p < 0.001. **B** Top 10 BPs, CCs and MFs in GO enrichment analysis. BP: biological process; CC: cellular component; MF: molecular function; **C** Top 30 KEGG pathways

Subsequently, we further analyzed the correlation between 16 DE-PRGs and immune cells in AS samples (Fig. 2A). The obtained results showed that some immune cells strongly correlated with DE-PRGs. For example, neutrophils were significantly negatively correlated with GZMA, GZMB, CYCS, and CHMP4A and significantly positively correlated with NOD2, NLRP1, NLRP4, IRF2, IRF1, and IL18. CD8 + T cells were significantly negatively correlated with NLRC4 and CHMP4A and significantly positively correlated with GZMA, GZMB, CYCS, and CHMP4A. Resting NK cells were significantly negatively correlated with CHMP4A and significantly positively correlated with GZMA and GZMB.

### Functional enrichment analysis of DE-PRGs

GO terms and KEGG pathways were used to determine the molecular biological functions of DE-PRGs in AS (Figs. 2B-C). In GO terms, positive regulation of cysteine-type endopeptidase activity involved in apoptotic process, pyroptosis, and positive regulation of interleukin-1 beta production was enriched in the biological process. The ESCRT III complex, amphisome membrane, and kinetochore microtubule were enriched in the cellular component category. Cytokine receptor binding, endopeptidase activity, and tumor necrosis factor receptor binding were enriched in the molecular function category. Regarding KEGG pathway analysis, DE-PRGs were significantly enriched in necroptosis, the NOD-like receptor signaling pathway, and the TNF signaling pathway.

### Screening of the key genes for creating the diagnostic model

**Fig. 3:**
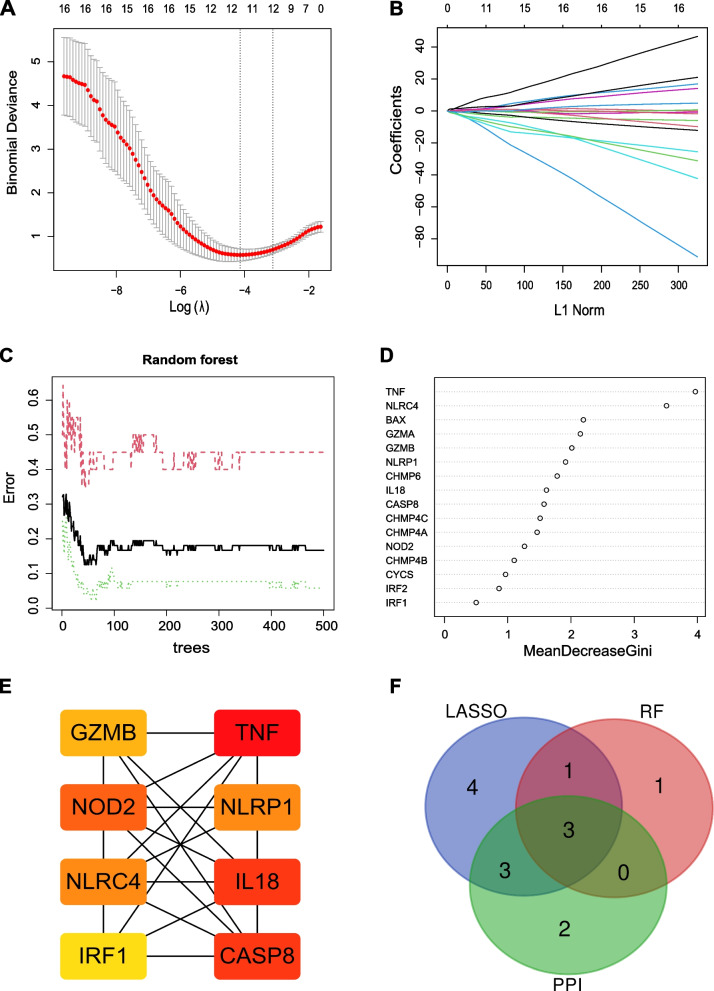
Screening for key genes based on machine learning and PPI network. **A**-**B** LASSO model. **C**-**D** Random forest model. **E** PPI network. **F** The intersections between the LASSO model, random forest model, and PPI network

The hub genes for constructing the AS diagnostic model were screened by PPI network, LASSO, and RF algorithms on 16 DE-PRGs. The results of the LASSO algorithm showed that 11 candidate genes were identified based on the optimal value of λ, including BAX, CHMP4B, CHMP4C, CHMP6, GZMB, IRF2, CASP8, NLRC4, NLRP1, NOD2, and TNF (Figs. 3A–B). For the RF model, the number of trees corresponding to the minimum error point was determined (number of trees = 43) (Fig. 3C), and candidate genes were obtained with a critical score of more than 2, including TNF, NLRC4, BAX, GZMA, and GZMB (Fig. 3D). The PPI network with 16 nodes and 41 edges was constructed based on STRING PPI confidence scores > 0.4 in the STRING platform (Supplementary file 4). The top 8 hub nodes were identified from the PPI network using the MCC method in cytoHubba (Fig. 3E), including TNF (score = 206), IL18 (score = 198), CASP8 (score = 198), NOD2 (score = 144), NLRP1 (score = 120), NLRC4 (score = 120), GZMB (score = 54), and IRF1 (score = 50). To guarantee the value of the diagnostic model, the intersection of LASSO, RF, and PPI was used as the best gene set for creating a diagnostic model (Fig. 3F). As a result, three genes (TNF, NLRC4, and GZMB) were identified and selected to develop a prediction signature.

### Construction and performance evaluation of the nomogram model

**Fig. 4:**
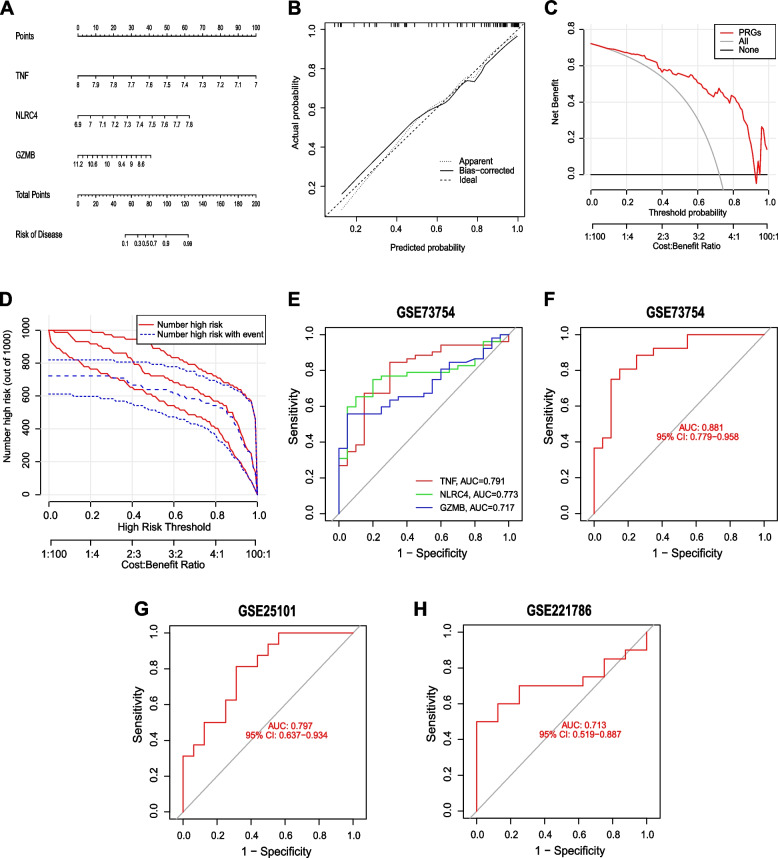
Construction and validation of the diagnostic model. **A** Construction of a nomogram for predicting AS risk based on 3 genes. The patients’ diagnostic model gene scores were summed to calculate the total score. The higher the total score, the higher the patient’s risk of being diagnosed with AS. **B**-**C** Construction of the calibration curve (**B**) and DCA (**C**) for assessing the predictive efficiency of the nomogram model. **D** Clinical impact curves. The red curve indicates the number of people classified as high risk by the model at the threshold probability for each variable; the blue curve is the number of true positives at the threshold probability for each variable. **E**–**H** ROC analysis of the 3-gene-based diagnostic model in the GSE73754 (**E**, **F**), GSE25101 (**G**), and GSE221786 (**H**) datasets

We created a nomogram using the three hub genes (TNF, NLRC4, and GZMB) (Fig. 4A). The calibration curve was used to estimate the predictive value of the nomogram, and the obtained results showed a high agreement between the predicted and the actual results (Fig. 4B). Subsequently, DCA and clinical impact curves were used to evaluate the clinical utility of the nomogram. The DCA showed that the nomogram had a high clinical net benefit at a threshold probability of 0.1–1.0 (Fig. 4C), and the clinical impact curves revealed remarkable predictive power for the nomogram (Fig. 4D). Finally, the predictive validity of the nomogram was confirmed using ROC curves (Figs. 4E-H). The AUC of the GSE73754 dataset was 0.881 (95% CI: 0.779, 0.958), the AUC of the GSE25101 dataset was 0.797 (95% CI: 0.637, 0.934), and the AUC of the GSE221786 dataset was 0.713 (95% CI: 0.519, 0.887). The ROC analysis results demonstrated that the nomogram model had high diagnostic performance.

### Consensus clustering of DE-PRGs identified two subtypes of AS

**Fig. 5:**
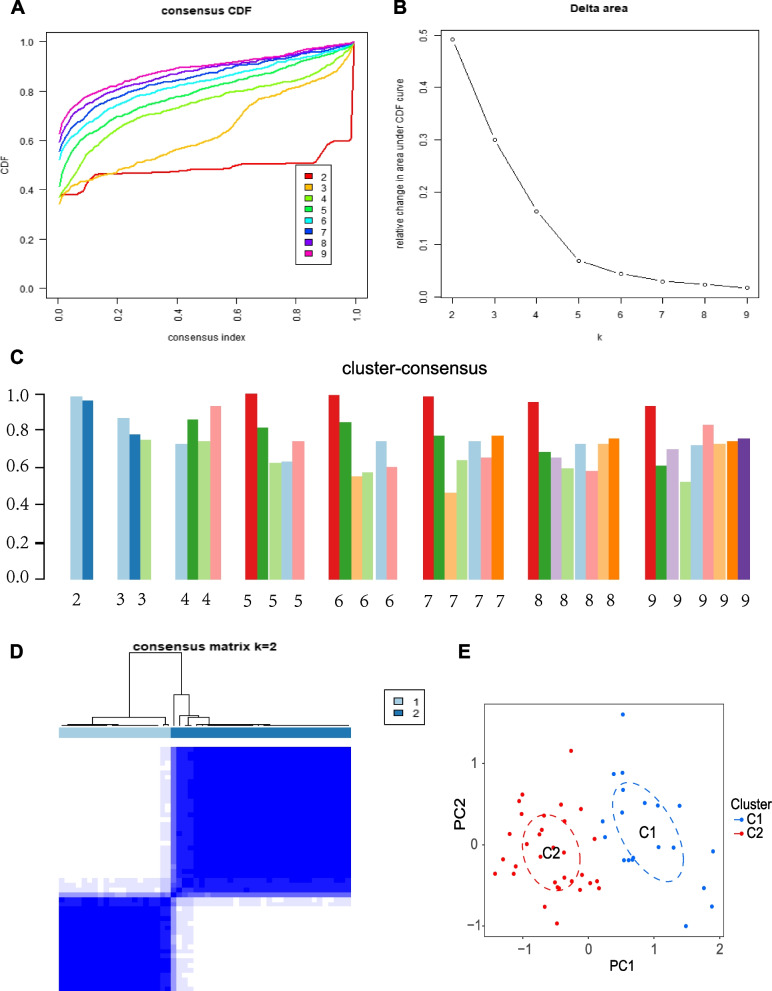
Identification of pyroptosis-related molecular clusters in AS. **A** Cumulative distribution function (CDF) curve. **B** CDF delta area. **C** Consensus clustering score. **D** Consensus clustering matrix when k = 2. **E** PCA visualizes the distribution of two clusters

The 16 DE-PRGs were used to screen the molecular subclusters of AS. The CDF value and Delta area showed that the clustering results were relatively stable when k = 2 (Figs. 5A-B). In addition, the consistency score of each subtype was > 0.9 only when k = 2 (Fig. 5C). Examination of the consensus matrix showed that k = 2 was the best option and that each sample in the cluster exhibited a strong correlation (Fig. 5D). Finally, the 52 AS samples were stratified into two distinct subtypes: Cluster 1 (C1) and Cluster 2 (C2). C1 contains 20 AS samples, and C2 involves 32 AS samples. Principal component analysis (PCA) indicated a significant difference between the two patterns (Fig. 5E).

### Expression of DE-PRGs and immune infiltration between the two pyroptosis subtypes

**Fig. 6:**
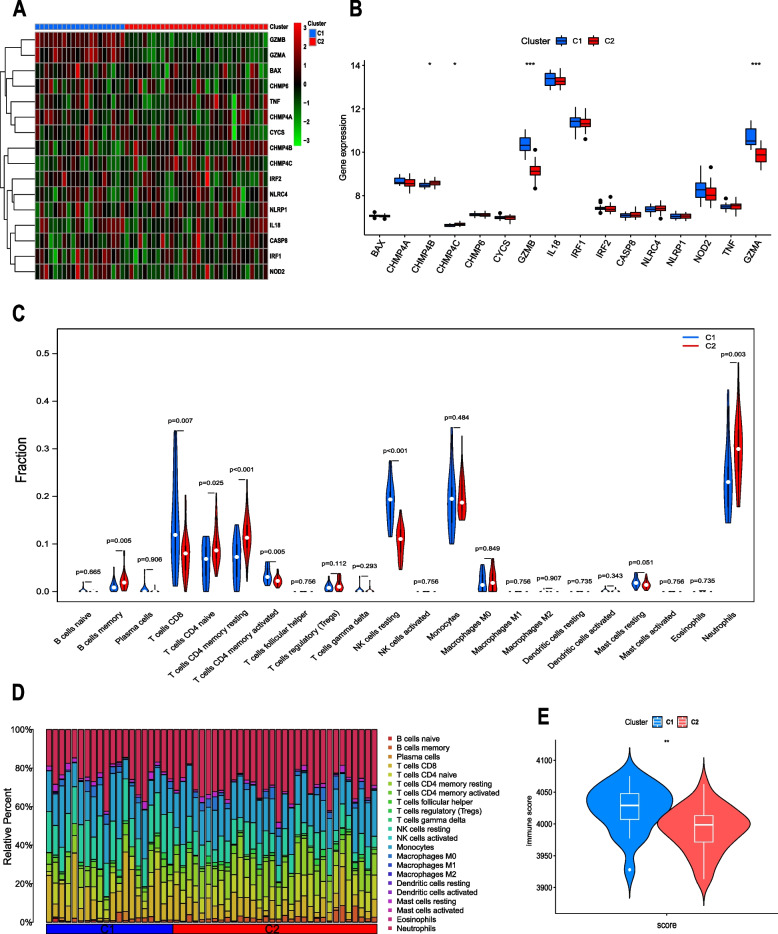
Expression of DE-PRGs and immune analysis between the two subtypes. **A** Heatmap showing the expression of the 16 DE-PRGs in the two pyroptosis subtypes. **B** Boxplot showing the expression of 16 DE-PRGs in two pyroptosis subtypes. *p < 0.05, ***p < 0.001. **C** Differences in immune infiltration between the two pyroptosis subtypes. **D** The relative infiltration abundance of immune cells between the two pyroptosis subtypes. **E** The estimated immune score between the two pyroptosis subtypes

The expression differences of 16 DE-PRGs between the two clusters were compared and are shown in the heatmap and boxplot (Figs. 6A-B). The obtained results showed that GZMA and GZMB were significantly upregulated in C1, and CHMP4B and CHMP4C were significantly upregulated in C2.

We also compared the differences in immune infiltration between the two subclusters. First, the ESTIMATE results indicated that C1 had a significantly higher overall immune score than C2 (Fig. 6E). Subsequently, we further analyzed the immune cell infiltration level and immune function level of the two subclusters. As expected, we observed that C1 had more immune cell infiltration and immune response. CIBERSORT analysis results showed that the infiltration level of CD8 T cells, activated memory CD4 + T cells, and resting NK cells in C1 was higher than that in C2, and the infiltration levels of memory CD4 + T cells, resting memory CD4 + T cells, naive CD4 + T cells and neutrophils in C1 were lower than those in C2 (Figs. 6C-D).

### WGCNA screening feature module

**Fig. 7:**
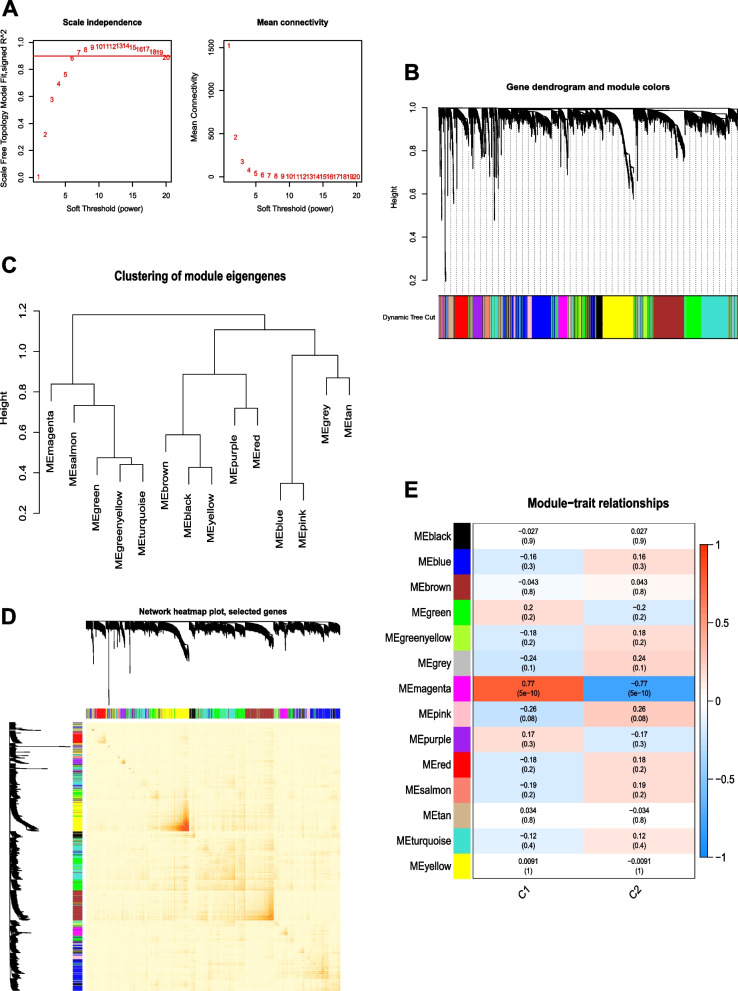
Coexpression analysis for two clusters. **A** The scale-free fit index and mean connectivity were used to select various soft threshold powers (β). **B** Dendrogram clustering of all genes between the two clusters was performed according to the topological overlap matrix (1-TOM). Each branch in the clustering tree represents a gene, and different colors represent different coexpression modules. **C** Clustering of module eigengenes. **D** Heatmap of the correlation between 14 modules; **E** Correlation between modules and clusters

WGCNA was used to identify the key gene modules between the two pyroptosis clusters in the 52 AS samples. First, 52 AS samples were clustered to detect and remove outliers, and a final sample of 45 was included for follow-up analysis (Supplementary file 5). Then, we set the soft-threshold power (β) to 6 and the scale-free (R^2^) to 0.9 (Fig. 7A). Fourteen modules were determined, which are represented by different colors (Figs. 7B-C). The TOM heatmap revealed the adjacency of ME (Fig. 7D). The module-trait correlation analysis showed that the magenta module (276 genes) had the highest correlation with two clusters (cor =  ± 0.77, *P* = 5e-10) (Fig. 7E).

### Enrichment analysis of the magenta module

**Fig. 8:**
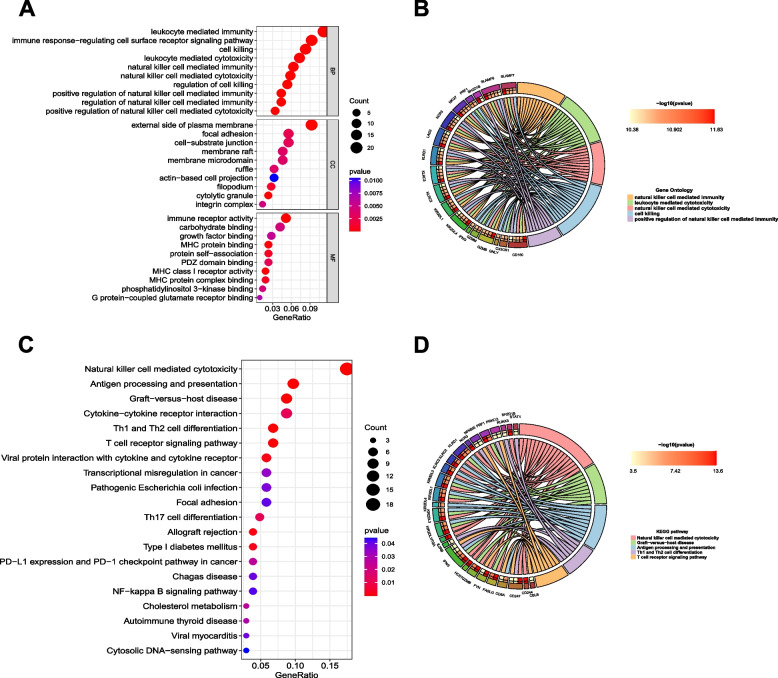
Enrichment analysis of the magenta module. **A** Top 10 BPs, CCs and MFs in GO enrichment analysis. BP: biological process; CC: cellular component; MF: molecular function. **B** The top 5 GO analysis results and their associated genes. **C** Top 30 KEGG pathways. **D** The top 5 KEGG pathways and their associated genes

GO and KEGG analyses were used to further explore the function and mechanism of the magenta module gene with the highest correlation with the pyroptosis clusters. Interestingly, most results were closely related to the immune response. In GO terms, leukocyte-mediated immunity, immune response-regulating cell surface receptor signaling pathway, and cell killing were enriched in the biological process category. External side of plasma membrane, focal adhesion, and cell-substrate junction were enriched in the cellular component. Immune receptor activity, carbohydrate binding, and growth factor binding were enriched in the molecular function category (Fig. 8A). The top five results were selected based on P values, showing the genes associated with them (Fig. 8B). KEGG analysis showed that 276 genes of the magenta module were mostly enriched in natural killer cell-mediated cytotoxicity, antigen processing and presentation, and graft-versus-host disease (Fig. 8C). The top five pathways were selected based on P values, showing the genes associated with them (Fig. 8D).

### Identify small-molecule drugs with potential therapeutic value

**Fig. 9:**
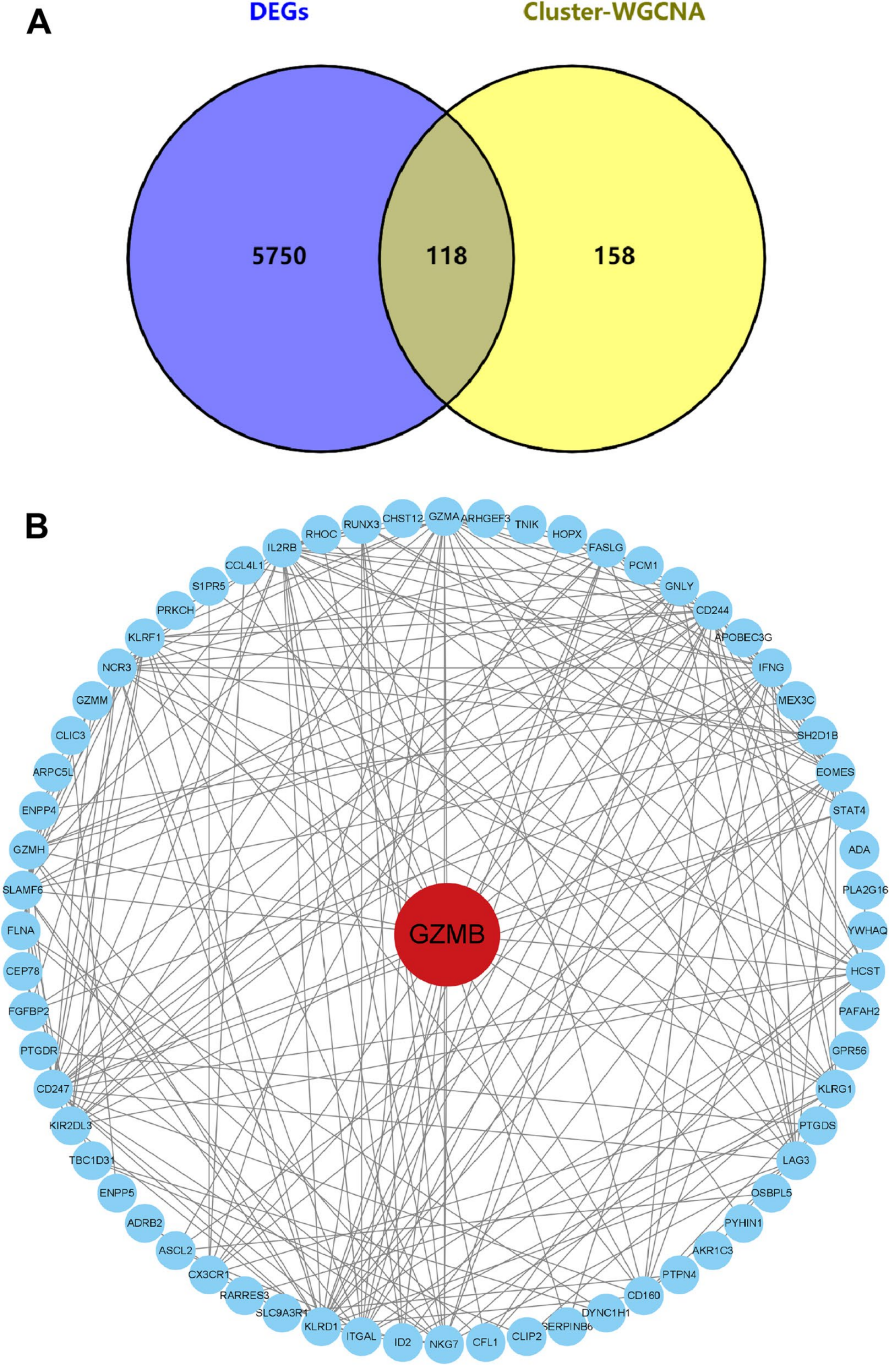
Identification of the hub gene. **A** The intersection of the DEGs of AS-control and the DEGs of subtypes. **B** PPI network

A total of 118 genes intersected between the magenta module gene and DEGs (AS vs. healthy controls), and these genes showed different expression levels in the AS-control and two pyroptosis subtypes (Fig. 9A). According to the expression of 118 genes in AS, they were divided into high expression and low expression groups. Subsequently, through the CMap online website, the top 3 small molecule drugs with opposite AS expression patterns, including ascorbic acid, RO 90–7501, and ryanodine, were screened based on their scores (Table 1). These drugs may be able to reverse the genetic changes in the pathogenesis of AS and serve as potential drugs for AS treatment.

**Table 1:** CMAP analysis

Name	Score	Description	PubChem ID
Ascorbic acid	-99.02	Antioxidant	54,670,067
RO 90–7501	-97.95	Beta amyloid inhibitor	824,226
Celastrol	-97.89	Anti-inflammatory	122,724

### Screening of hub genes and molecular docking

**Fig. 10:**
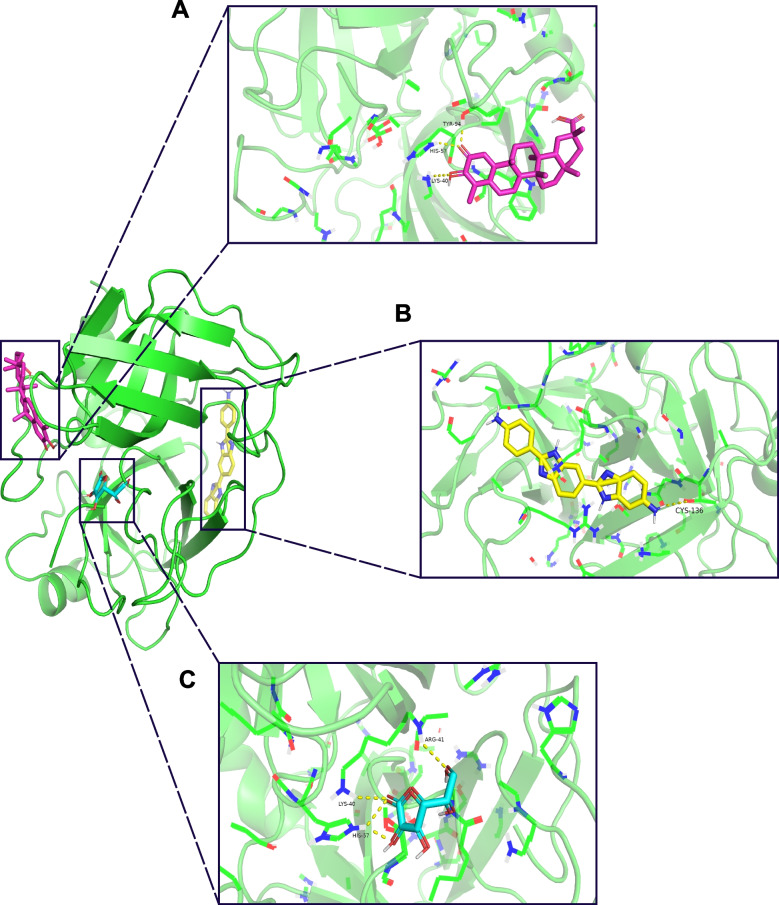
Molecular docking results. **A** A 3D view of the best-selected conformation of GZMB and celastrol. **B** A 3D view of the best-selected conformation of GZMB and RO 90–7501. **C** A 3D view of the best-selected conformation of GZMB and ascorbic acid. Green represents protein

We used PPI to identify the most critical gene from 118 genes. After obtaining the PPI network with the same method as above, we used cytoHubba to calculate the score of each gene. We tried the four most commonly used algorithms (MCC, MNC, EPC, and DEGREE) (Supplementary file 6). Surprisingly, three algorithms obtained consistent results, with GZMB obtaining the highest score (MCC = 535,352, MNC = 26, DEGREE = 26) (Fig. 9B). The protein structure of GZMB was obtained from the PDB database (PDB ID: 1IAU), and the docking pattern with the lowest binding affinity (kcal/mol) was visualized with PyMOL (Supplementary files 7, 8 and 9). The obtained results showed that GZMB and celastrol formed three hydrogen bonds, including TYR-94, HIS-57, and LYS-40 (affinity: -9.4 kcal/mol) (Fig. 10A). GZMB and RO-90–7501 formed one hydrogen bond, including CYS-136 (affinity: -8.8 kcal/mol) (Fig. 10B). GZMB and ascorbic acid formed three hydrogen bonds, including ARG-41, LYS-40, and HIS-57 (affinity: -5.3 kcal/mol) (Fig. 10C).

## Discussion

AS is a chronic inflammatory autoimmune disease, and the diagnosis and treatment of AS have been limited because its pathogenesis is still unclear [31]. Therefore, exploring the underlying mechanism of AS initiation and progression is important and urgent. Recent studies have investigated additional genes and inflammatory biomarkers associated with the pathogenesis of AS. Ye et al. [32] found that serum from AS patients induced mitochondrial dysfunction in mesenchymal stem cells in vitro. Notably, altered mitochondrial morphology and function is a major feature of ferroptosis and an important way to distinguish it from other regulatory cell death [33]. Rong et al. [34] found that iron death may affect AS through potential molecular regulatory pathways. In addition, ribosome-related genes and N6-methyladenosine RNA methylation regulators were also found to be dysregulated in AS [35, 36].

Pyroptosis is a proinflammatory type of cell death that releases proinflammatory cytokines and immune substances through cell rupture [37]. Excessive release of inflammatory factors triggers an overactive immune system and leads to the progression of autoimmune diseases [38]. There is now increasing evidence that pyroptosis plays an integral role in the pathogenesis of autoimmune diseases. Studies have shown that the expression of the pyroptosis-associated inflammasome is increased in the salivary glands of Sjogren’s Syndrome (SS) patients, and the number of salivary glands epithelial cells in SS patients is reduced by pyroptosis, resulting in a significant decrease in salivary secretion [39, 40]. Anti-double-stranded DNA antibodies, the hallmark antibody of systemic lupus erythematosus (SLE), have been found to bind to TLR4, which in turn activates NLRP3 inflammasome [41]. In addition, Pentaxin 3, which is upregulated in the plasma of patients with rheumatoid arthritis, can act synergistically with the ligand C1q to activate NLRP3 inflammasome, causing caspase-1-mediated pyroptosis and inflammatory cytokines, the degree of which was consistent with disease activity [42]. However, the relationship between pyroptosis and AS remains unclear. Studying the potential association between AS and pyroptosis will provide a foundation for diagnosis and clinical treatment. Therefore, this study was the first to systematically analyze the role of pyroptosis in AS and its immune microenvironment and constructed an AS diagnostic model by key DE-PRGs. In addition, we further detected the molecular subtypes of AS from the perspective of pyroptosis and analyzed the characteristics of different subtypes, which will provide new insights for individualized treatment of AS.

In this study, we performed differential expression analysis of 52 PRGs with data from the GSE73754 dataset. The obtained results showed that the expression of 16 PRGs was significantly different between AS samples and healthy controls. Subsequently, we performed some immune infiltration analyses. We first analyzed the infiltration abundance of immune cells in AS and healthy samples. Notably, according to the results, a significant increase in neutrophil infiltration and a significant decrease in CD8 + T cell infiltration were observed in AS samples, which is consistent with the results of previous studies [43]. We further analyzed the correlation between 16 DE-PGRs and immune cells in AS samples. The results showed significant positive or negative correlations between DE-PGRs and immune cells, such as neutrophils, CD8 + T cells, and resting NK cells. Innate immunity plays an important role in the pathological progression of AS, of which neutrophils are an essential cell type [44]. Recent studies suggest that neutrophils may play an important role in maintaining autoinflammation and autoimmunity by releasing neutrophil extracellular traps (NETs) carrying bioactive molecules [45]. Jiang et al. [46] suggest that upregulated neutrophils may be a key factor in the progression of AS. CD8 + T cells, antigen-presenting targets of HLA-B27 molecules, are considered to be involved in the occurrence and development of AS [47]. In AS patients, the inhibitory receptor for NK cells, KIR3DL1, interacts with multiple subtypes of HLA-B27, thereby inhibiting NK cell activity [48]. Moreover, innate immune cells can play an essential role in the pathogenesis of AS by secreting cytokines, such as IL-17, IL-22, IL-23, IL-1β, and TNF-α [1]. We further analyzed the correlation between 16 DE-PRGs and found clear synergistic or antagonistic effects among some DE-PRGs, suggesting that these DE-PRGs may play a role in AS through interactions. In summary, pyroptosis may affect the immune status of AS, and thus the progression of AS, and the abnormal expression of pyroptosis-related genes may be the beginning of autoimmunity and chronic inflammatory responses in AS, but the underlying mechanism of inducing the abnormal expression of pyroptosis genes in AS remains to be further elucidated. Our study will help to explore the mechanism of the occurrence and development of AS.

Based on the 16 DE-PRGs, we used PPI, LASSO, and RF to screen candidate genes. The intersection of the three algorithms was identified as the hub gene for building the AS diagnostic model. Three genes (TNF, NLRC4, and GZMB) were ultimately screened. TNF encodes a multifunctional proinflammatory cytokine that is involved in regulating various biological processes, including cell proliferation, differentiation, and apoptosis [49]. In addition, this cytokine is closely related to the pathological mechanisms of various diseases, including autoimmune diseases, AS, and cancer [50]. Currently, TNFi has been widely used in patients with AS [51]. TNFi dramatically improved symptoms by blocking TNF cytokines, which play an important role in inflammation [52]. NLRC4 is a member of the nucleotide binding and oligomerization domain (NOD)-like receptor (NLR) family [53]. NLRC4 can induce proinflammatory cytokine maturation and pyroptosis by activating caspase-1. Current studies have shown that NLRC4 is widely involved in regulating immune responses and plays an important role in metabolic diseases, tumors, and autoimmune diseases [54]. GZMB is a member of the peptidase S1 family of serine proteases that plays an important role in tissue healing, chronic inflammation, and the immune response [55]. Noticeably, GZMB is closely associated with NK cell activity. A recent study showed that patients with AS exhibited NK cell depletion and downregulated GZMB and GZMA expression compared to normal controls [56]. This is consistent with the results of this study, our results showed that GZMA and GZMB exhibited significant synergistic effects and were downregulated in AS samples. In addition, immune cell correlation analysis showed that GZMA and GZMB were significantly positively correlated with resting NK cells and negatively correlated with neutrophils. Finally, we constructed nomograms based on TNF, NLRC4, and GZMB and validated the diagnostic value of the model in two datasets. The obtained results showed that the actual result in the calibration chart was highly consistent with the predicted result, suggesting that the model could provide a valuable reference for the prediction of AS, and the DCA and clinical impact curves indicated that the model had significant clinical utility. The ROC curve indicated that the model had good diagnostic value. Thus, combined with previous reports and our findings, the potential value of this model in clinical applications is fully demonstrated.

Based on the 16 DE-PRGs, we identified two pyroptosis clusters. First, we assessed the expression levels of 16 DE-PRGs between the 2 clusters, and the obtained results showed that CHMP4B and CHMP4C were downregulated in C1, and GZMA and GZMB were downregulated in C2. Subsequently, we used ESTIMATE and COBERSORT to assess the immune status of the two pyroptosis clusters. The two algorithms obtained roughly similar results. We found that C1 had a higher total immune infiltration score and mediated more immune cell infiltration. Thus, C1 may represent a more immunoreactive microenvironment, and C2 represents an immunosuppressive microenvironment. Moreover, we noted that the level of neutrophil infiltration in C2 was significantly higher than that in C1, suggesting that a more severe inflammatory response may exist in C2. If dead neutrophils are not cleared in time, tissue damage will be aggravated. Meanwhile, according to previous studies, cytokines and inflammatory mediators produced during the inflammatory response can recruit or reject immune cell subpopulations by establishing gradients and ultimately promote the formation of an immunosuppressive microenvironment and immune escape [57–59]. Then, we further identified the signature gene module (magenta module) of C1 and C2 using WCGNA and performed enrichment analysis of the signature module genes. GO analysis showed that the signature module genes are involved in immune regulation and associated with various immune cell-mediated immune functions. Similarly, the results of KEGG analysis suggest that magenta module genes are involved in molecular pathways mostly related to immunity, such as the differentiation of Th1 and Th2 cells and the NF-κB signaling pathway. The Th1 immune response is proinflammatory, and its overactivation will recognize self-antigens and generate autoimmunity; the Th2 immune response is anti-inflammatory and has the effect of counteracting the Th1 immune response. The magenta module gene plays an important role in the development of AS by regulating the differentiation of Th1 and Th2 cells. The NF-κB signaling pathway, one of the critical regulatory pathways in the inflammatory and immune responses, has also been reported to be involved in the pathological progression of AS [60]. In conclusion, based on the characteristics of the two pyroptosis clusters, the powerful ability of different pyroptosis clusters to discriminate immune phenotypes was confirmed.

We intersected the magenta module genes with DEGs (AS vs. healthy control) and obtained a total of 118 intersection genes. Then, the PPI network was used to identify the most critical genes. We tried the four most commonly used algorithms, including MCC, MNC, EPC, and DEGREE, and the gene with the highest scores in the four results was GZMB. Notably, GZMB is also one of the three key genes for which we constructed the AS diagnostic model above. Meanwhile, the expression of GZMB in AS was also verified in previous experimental studies. Therefore, GZMB may be the hub gene for distinguishing AS from normal controls and different pyroptotic subtypes of AS.

We divided 118 intersecting genes into upregulated and downregulated groups according to their expression in AS, uploaded them to CMap, and finally obtained three small-molecule preparations (ascorbic acid, RO-90–7501, and celastrol). Ascorbic acid, also known as vitamin C, plays an important role as an antioxidant or cofactor for various enzymatic reactions in the prevention and treatment of many diseases, such as cancer, infectious diseases, and cardiovascular diseases [61]. Moreover, ascorbic acid exists in large quantities in immune cells and has key functions, such as maintaining immune balance and reducing proinflammatory responses. Studies have shown that ascorbic acid may regulate the cell death process of neutrophils, which can reduce the proportion of neutrophil necrosis and cause more neutrophils to develop apoptosis while accelerating the evacuation of dead neutrophils at the site of inflammation. Therefore, ascorbic acid can effectively reduce the inflammatory response and tissue damage [62–64]. AS, an inflammatory autoimmune disease, has higher levels of neutrophil infiltration than healthy controls. Ascorbic acid may inhibit the pathological progression of AS by acting on the regulatory processes of neutrophils. In addition, our findings suggest that the abundance of neutrophil infiltration is significantly higher in C2 than in C1. Ascorbic acid may have a better therapeutic effect on C2. RO 90–7501 acts as an inhibitor of amyloid β (Aβ) protofibril assembly and reduces Aβ-induced cytotoxicity [65]. We note that previous studies have shown that Tg2576 transgenic mice with high expression of Aβ have abnormal bone metabolism, and Aβ may regulate osteoclast activity in an age-dependent manner, resulting in increased bone resorption and inhibition of osteoblast differentiation, eventually leading to osteoporosis and decreased bone reconstruction ability [66, 67]. In addition, elevated levels of Aβ and amyloid precursor protein have been observed in human osteoporotic vertebral trabecular bone and femoral neck specimens [68]. Therefore, Aβ may be one of the regulators of bone metabolism, and abnormal expression of Aβ may have important effects on bone development and metabolism, as well as bone formation and resorption. Osteoporosis is a common complication of AS and one of the critical causes of spinal fractures. Most patients present with decreased bone density and abnormal bone metabolism. RO 90–7501 may improve the prognosis of AS patients by regulating bone metabolism by inhibiting Aβ expression. Celastrol, a bioactive molecule extracted from Thunder God Vine, has shown great potential in treating various inflammatory and autoimmune diseases, such as arthritis, inflammatory bowel disease, and systemic lupus erythematosus, through the modulation of immune cell signaling in the inflammatory microenvironment [69]. Currently, celastrol’s potential pharmacological effects and application value are being discovered in an increasing number of diseases, but studies on AS are still minimal [70, 71]. One study showed that celastrol could inhibit the cell proliferation of isolated AS fibroblasts and in vitro osteogenic differentiation [72], suggesting that celastrol potentially affects bone metabolism in AS patients. In summary, these three drugs may have potential therapeutic value for AS and may even have different therapeutic effects for different pyroptosis subtypes of AS. Finally, we used molecular docking to verify the binding ability of the three drugs to the hub protein (GZMB), and the obtained results showed that RO 90–7501 (affinity: -8.8 kcal/mol) and celastrol (affinity: -9.4 kcal/mol) achieved satisfactory results. We infer that RO 90–7501 and celastrol may inhibit the pathological progression of AS by acting on GZMB proteins and regulating pyroptosis. However, more experimental evidence is needed in future studies for validation.

This study has some limitations and shortcomings. First, this study used only retrospective data from the GEO database, and prospective studies are needed to confirm these findings. Second, the conclusions reached through data analysis in this study need to be further confirmed by more experiments.

## Conclusions

In conclusion, our study revealed that pyroptosis might play an essential role in regulating the immune microenvironment of AS, and pyroptosis-related genes showed excellent discrimination for AS. Moreover, we identified drugs with potential therapeutic value for AS by CMap and molecular docking. Our study will provide new insights into the molecular mechanism, diagnosis, and treatment of AS.

## Supplementary Information

**Additional file 1: Supplementary file 2.** List of pyroptosis genes.

**Additional file 2: Supplementary file 3.** Immune analysis. (A) The relative infiltration abundance of immune cells between AS and control. (B) Differences in immune infiltration between AS and control.

**Additional file 3: Supplementary file 4.** PPI network of 16 DE-PRGs.

**Additional file 4: Supplementary file 5.** Detection of outliers and rejection.

**Additional file 5: Supplementary file 6.** Calculation of the highest scoring gene using cytoHubba.

**Additional file 6: Supplementary file 7.** Results of 20 molecular docking studies between celastrol and GZMB.

**Additional file 7: Supplementary file 8.** Results of 20 molecular docking studies between RO 90-7501 and GZMB.

**Additional file 8: Supplementary file 9.** Results of 20 molecular docking studies between ascorbic acid and GZMB.

## Data Availability

The datasets analysed during the present study are available in the GEO repository [GSE73754, https://www.ncbi.nlm.nih.gov/geo/query/acc.cgi?acc=GSE73754], [GSE25101, https://www.ncbi.nlm.nih.gov/geo/query/acc.cgi?acc=GSE25101], [GSE221786, https://www.ncbi.nlm.nih.gov/geo/query/acc.cgi?acc=GSE221786].

## Abbreviations

AS: Ankylosing spondylitis
PRG: Pyroptosis-related gene
DEG: Differentially expressed gene
DE-PRG: Differentially expressed pyroptosis-related gene
GEO: Gene Expression Omnibus
GO: Gene ontology
KEGG: Kyoto Encyclopedia of Genes and Genomes
PCA: Principal component analysis
PPI: Protein–protein interaction
CMAP: Connectivity map
TNFi: Tumor necrosis factor inhibitors
LASSO: The least absolute shrinkage and selection operator
RF: Random forest
DCA: Decision curve analysis
AUC: The area under the curve
CDF: Consensus cumulative distribution function
WGCNA: Weighted gene correlation network analysis
TOM: Topological overlap matrix
ME: Module eigengene
Aβ: Amyloid β
