# Supplementary figures and images for: Exploring hub pyroptosis-related genes, molecular subtypes, and potential drugs in ankylosing spondylitis by comprehensive bioinformatics analysis and molecular docking
Source: BMC Musculoskelet Disord. 2023 Jun 29;24:532. doi: 10.1186/s12891-023-06664-8 (PMC10308648; doi:10.1186/s12891-023-06664-8)

**A**

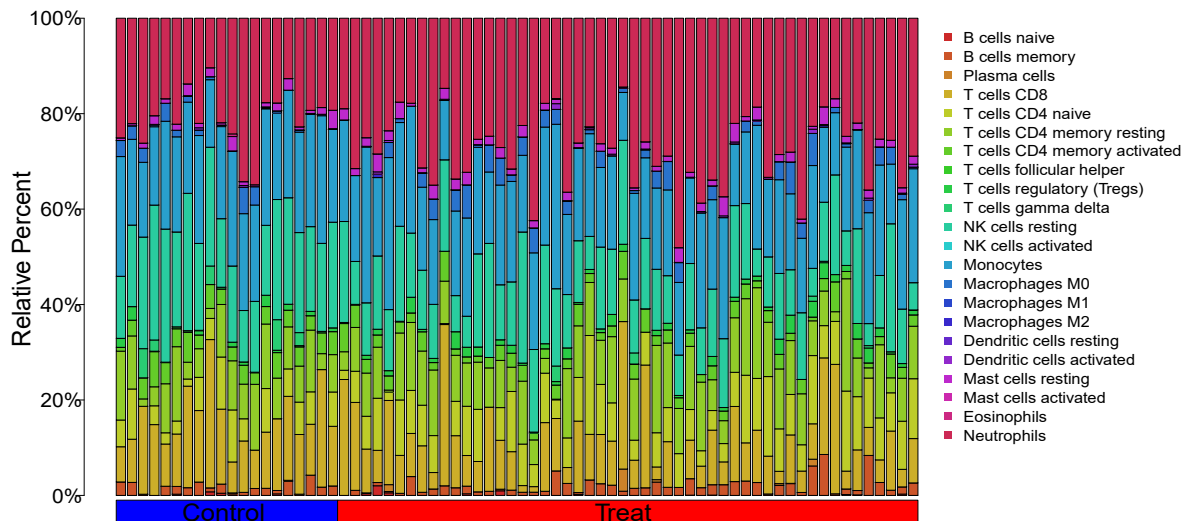

**B**

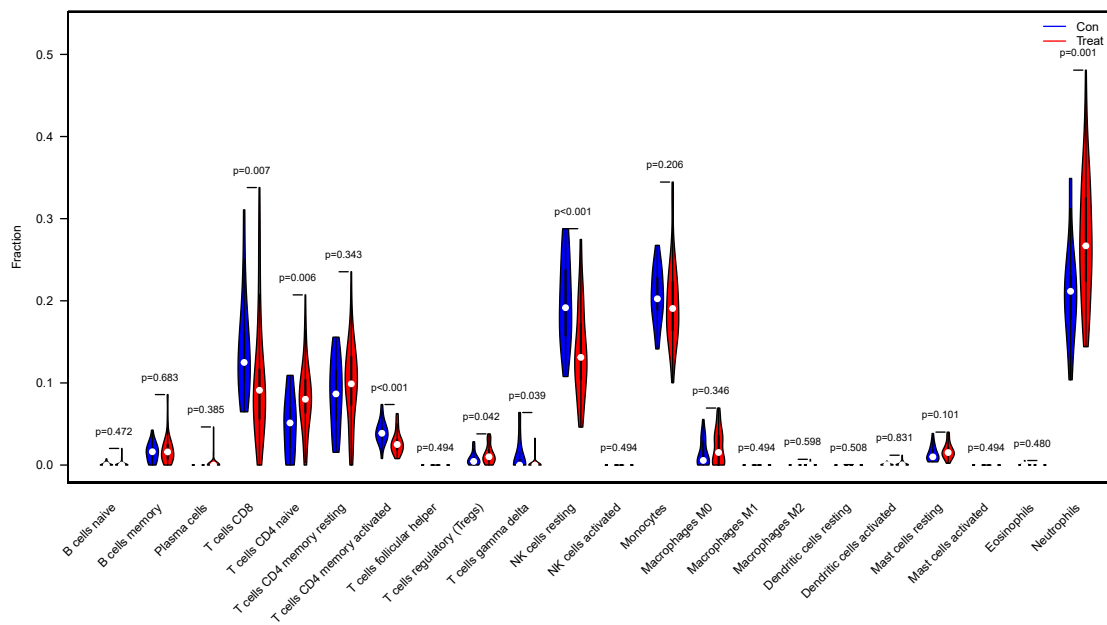

Supplement: Supplementary file 2 — Additional file 2: Supplementary file 3. Immune analysis. (A) The relative infiltration abundance of immune cells between AS and control. (B) Differences in immune infiltration between AS and control. [file 12891_2023_6664_MOESM2_ESM.pdf]

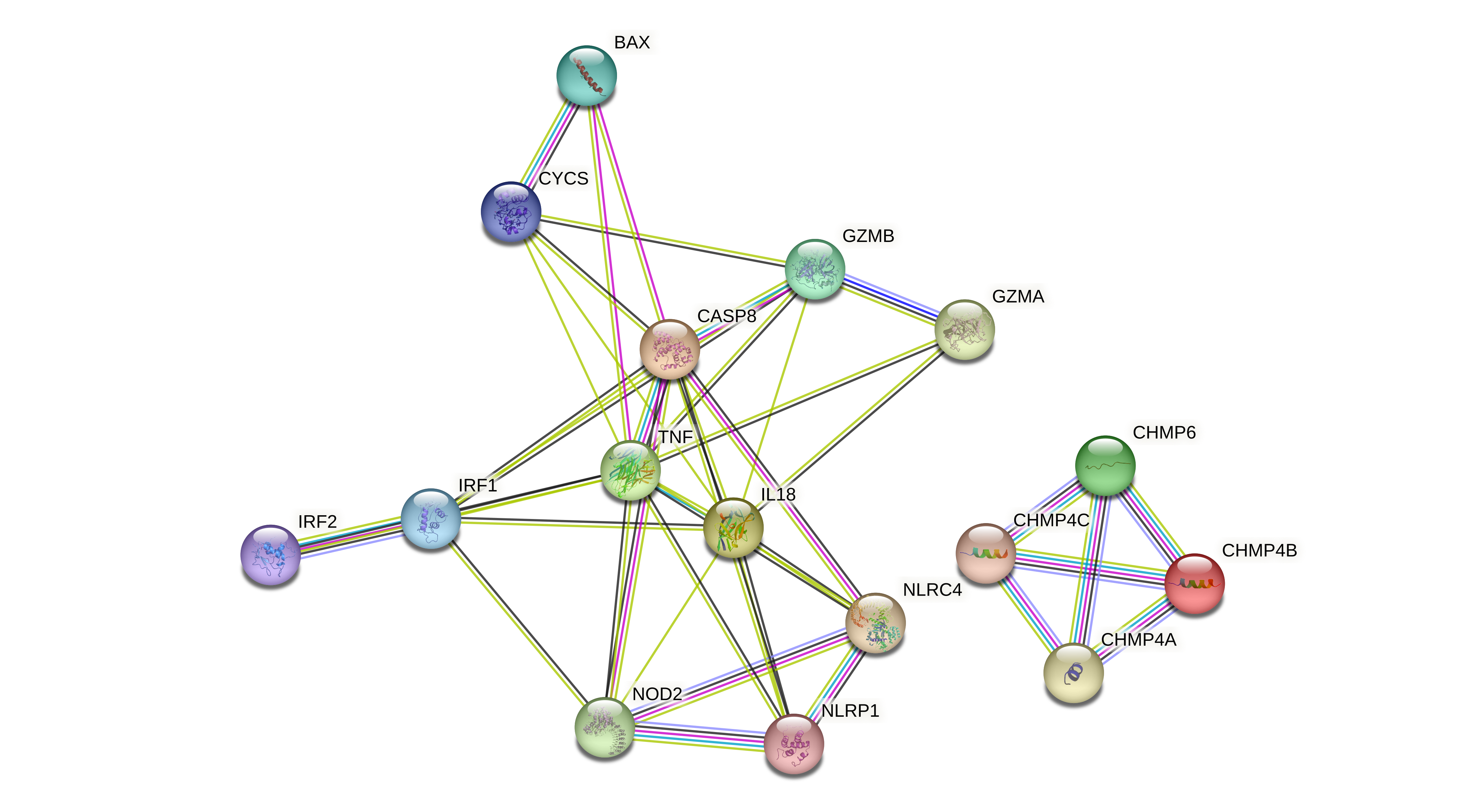

Supplement: Supplementary file 3 — Additional file 3: Supplementary file 4. PPI network of 16 DE-PRGs. [file 12891_2023_6664_MOESM3_ESM.png]

# Sample clustering to detect outliers

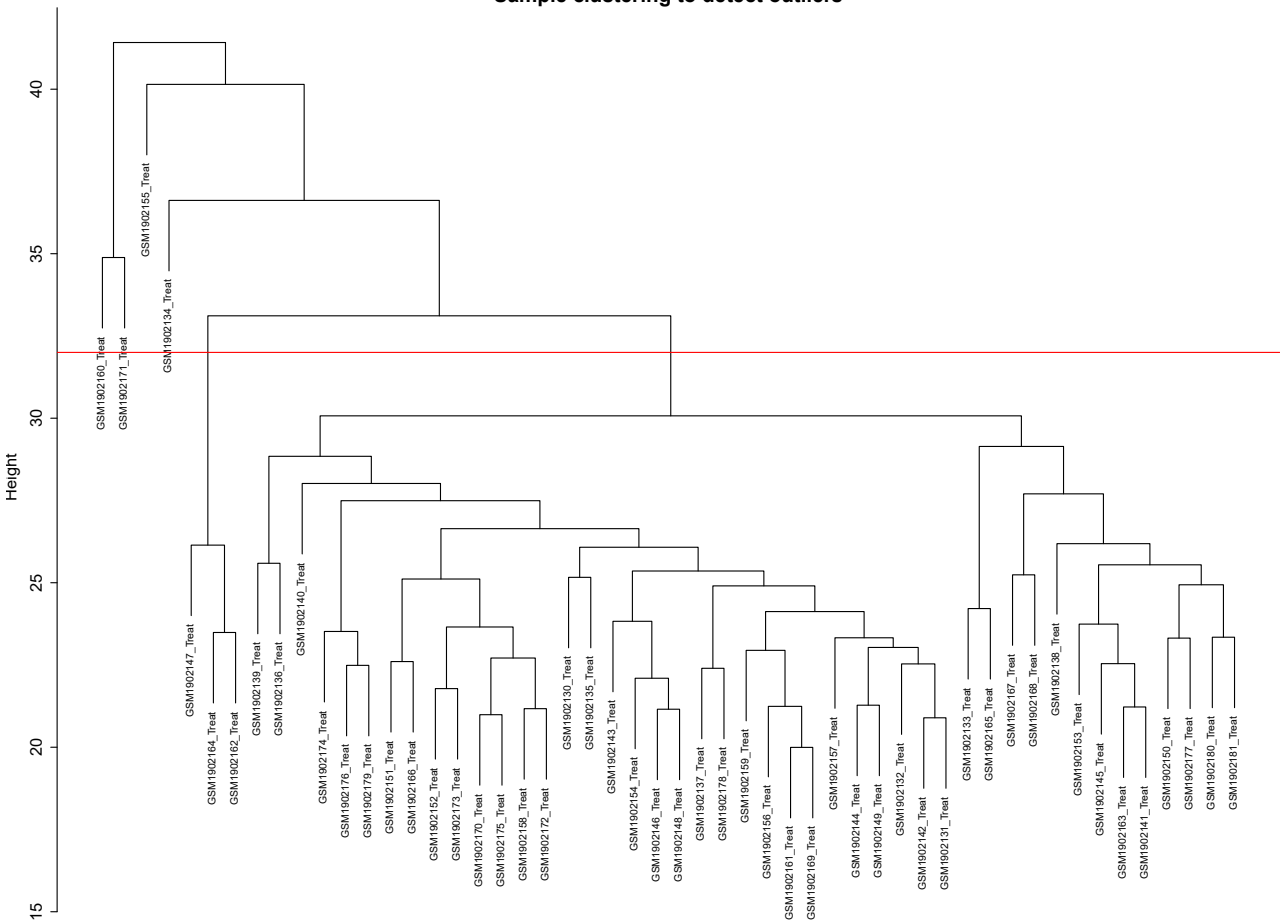

Supplement: Supplementary file 4 — Additional file 4: Supplementary file 5. Detection of outliers and rejection. [file 12891_2023_6664_MOESM4_ESM.pdf]
